# Disentangling the effects of task difficulty and effort on flow experience

**DOI:** 10.1007/s00426-025-02128-x

**Published:** 2025-06-26

**Authors:** Hairong Lu, Dimitri Van der Linden, Arnold B. Bakker

**Affiliations:** 1 Center of Excellence for Positive Organizational Psychology, Erasmus University Rotterdam, Rotterdam, The Netherlands; 2 School of Management, Human Resource Management, University of Vaasa, Vaasa, Finland; 3 Department of Industrial Psychology and People Management, University of Johannesburg, Johannesburg, South Africa

## Abstract

Flow, which is a rewarding state of full focus, typically arises when engaging in tasks with an optimal level of difficulty that is matched with a person’s skill level. Meanwhile, optimal task difficulty usually comes with the greatest effort exertion. We propose that not only the difficulty level, but also the level of effort invested plays a role in the subjective feelings of flow. Using a visual discrimination task, we manipulated stimulus complexity and the expected probability of detecting a difference to induce and disentangle perceived task difficulty and effort exertion, respectively. Notably, perceived task difficulty increased proportionally with increasing stimulus complexity. Reaction time as an index of effort exertion, increased in challenging tasks with higher expectancy. Patterns of flow experience mirrored the observed shifts in effort exertion, suggesting a possible link between flow and effort. However, no parallel trend emerged in the physiological flow indicator, specifically the P300 amplitude. These findings highlight the intricate interplay between subjective experiences of task difficulty, exerted effort, and the subjective sense of being in ‘flow’.

**Supplementary Information:**

The online version contains supplementary material available at 10.1007/s00426-025-02128-x.

## Introduction

The concept of flow, a state characterized by deep concentration and intrinsic motivation (Csikszentmihalyi, 2000), has been extensively studied in various domains, including sports, arts, media, and at the workplace. Flow is considered a desirable state due to its link with positive affect, optimal performance and enhanced well-being (Bakker et al., 2011; Engeser & Rheinberg, 2008).

According to flow theory, individuals are more likely to enter a flow state when the challenge of a task aligns with the level of personal skills, resulting in a narrow flow band between challenge and skills. In line with this, there is an inverted U-shaped flow curve across increasing task difficulty (Csikszentmihalyi, 2000; Engeser & Rheinberg, 2008; Lu et al., 2023; Peifer et al., 2014). In the peak of the inverted U-shape, people are optimally engaged which implies that their attentional resources are maximized towards the task at hand. One relevant question, however, is whether the peak engagement and attention during flow automatically follows from the typical challenge-skills match, or whether it is the result from optimal levels of effort exerted.

More specifically, a separate body of research on mental effort suggests that individuals exert their highest level of effort on tasks that are challenging, yet still within their capabilities (Silvestrini et al., 2023; Verguts et al., 2015). When tasks become either too easy or overly difficult, there tends to be a decrease in effort. Accordingly, it is conceivable that the level of flow experience may not be fundamentally inherent to the challenge-skill match but may be linked with the amount of effort people put into the tasks.

Effort exertion, generally defined as the mobilization of resources to carry out instrumental behavior (Gendolla & Wright, 2009), plays a crucial role in the way individuals approach tasks. According to the energy conservation theory (Ligneul et al., 2022), individuals tend to conserve their energy and resources in less favorable situations, thereby impacting their motivation to invest effort. Previous research has highlighted a non-monotonic relationship (inverted-U shape) between task difficulty and multiple physiological indicators of effort allocation (Silvestrini et al., 2023; Verguts et al., 2015). In this context, individuals tend to invest the highest effort in tasks that are challenging yet feasible, enabling them to utilize their skills optimally and yield greatest learning progress (Kaplan & Oudeyer, 2007; Sayalı et al., 2023). However, when a task becomes excessively difficult, individuals are inclined to withdraw effort, which is accompanied with a decline in motivation and overall performance (Embrey et al., 2023; Patzelt et al., 2019).

Converging evidence highlights the striking similarities between effort exertion and the flow experience. For example, both effort exertion and flow experience exhibit inverted U-shaped relationships with task difficulty and both are influenced by individuals’ perceived task importance (Nakamura & Csikszentmihalyi, 2014; Silvestrini et al., 2023). Moreover, both effort exertion and flow experience have been demonstrated to be linked to pupil dilation (Lu et al., 2023; Van der Wel & van Steenbergen, 2018), suggesting a similar physiological response during the two states. In addition, effort exertion is linked to multiple important characteristics of the flow experience, e.g. the rewarding property, good attentional quality, and better performance (Bogdanov et al., 2022). Flow is usually also being depicted as a rewarding experience (Nakamura & Csikszentmihalyi, 2014). Despite effort being potentially aversive for individuals, expected value of control theory states that under some circumstances effort is being valued and even searched for (Clay et al., 2022; Frömer et al., 2021; Inzlicht et al., 2018; Shenhav et al., 2013). Empirical research further shows that cognitive effort exertion can lead to increased subsequent reward processing, as evidenced by elevated neural response to gains and losses in trials requiring more effort (Bogdanov et al., 2022). This supports the notion of a positive link between flow and effort exertion in terms of the rewarding nature. Moreover, there is a clear positive association between effort exertion and attentional quality, indicating that increased effort leads to a reduction in attentional lapses (Unsworth et al., 2022). Feelings of flow are typically accompanied with the mobilization of attentional resources (de Sampaio Barros et al., 2018), which fits with observations of extreme task focus in flow. Therefore, in general, the positive influence of effort exertion on subsequent reward processing, attentional quality and performance highlights its potential benefits for achieving flow.

Considering the parallel patterns observed between flow and effort exertion, it is conceivable that effort exertion plays a relevant role in facilitating the flow experience. However, the intertwined effects of task difficulty and effort exertion pose a significant challenge for researchers aiming to understand the underlying mechanisms of flow. Consequently, in all previous experimental studies on flow it is difficult to disentangle effects of effort from task difficulty and to assess their relative contribution. This limits our understanding of flow. To address this previous limitation, the present study aims to disentangle the mixed effects of task difficulty and effort exertion on perceived flow.

To isolate the influence of effort exertion from task difficulty on flow, we introduced a visual discrimination task. In this task, difficulty levels can be easily manipulated by varying image complexity, a well-established approach in the literature (Caroux & Mouginé, 2022; Rosenholtz et al., 2007). Building on the Expected Value of Control (EVC) theory (Shenhav et al., 2013), we introduced a novel manipulation -Expected Probability of Detecting a Difference (EPDD)- to influence effort exertion without affecting task difficulty. The EPDD refers to a participant’s subjective expectation about the likelihood of successfully identifying a difference between two visual stimuli in a discrimination task. According to EVC theory, individuals integrate information about the anticipated rewards and the likelihood of success to estimate the value of exerting cognitive control. This estimation guides the allocation of mental effort (Frömer et al., 2021). In the visual discrimination task, participants detect subtle differences between two images through a series of saccades. These saccades allow individuals to systematically compare specific regions of each image, gathering detailed visual information to identify discrepancies (Wang et al., 2024). Once sufficient information is collected to make a decision (e.g., spotting a difference), further saccades become unnecessary. Therefore, each trial concludes with either successfully identifying a difference or confirming the absence of differences. And detecting a difference is likely tied to a sense of accomplishment and being favored by individuals as it is aligned with their goal.

According to prior research (Lewis and Simmons, 2020), higher probabilities of favorable outcomes encourage greater effort exertion, while lower probabilities reduce motivation. In the visual discrimination task context, detecting a difference may be favorable because it provides tangible, rewarding outcomes that align with participants’ goals and thereafter influence the level of effort participants invest in searching for discrepancies. Therefore, by manipulating participants’ expectations about the probability of detecting differences, we modulated their willingness to exert effort. This manipulation allowed us to decouple effort exertion from task difficulty, providing valuable insights into the mechanisms underlying optimal engagement and performance in challenging tasks. We hypothesize that, for a given task, individuals will exert more effort in conditions where they hold a higher EPDD compared to those with a low expectancy, and flow is expected to vary proportionally with effort exertion. Importantly, we anticipate that this effect will be most pronounced in relatively difficult tasks in which, due to the complexity of image, participants have more uncertainty about whether there indeed is a difference or no differences between the images (Embrey et al., 2023; Patzelt et al., 2019). In such tasks, the heightened effort driven by higher EPDD is likely to play a pivotal role in sustaining engagement and facilitating the flow state, whereas in easier tasks, the impact of expectancy on effort and flow may be less substantial due to the lower cognitive and motivational demands (Kraus et al., 2024). In simple images, there is often less uncertainty about similarity, which means that EPDD may have a minimal impact on effort exertion in easy tasks.

In addition to examining the effects of task difficulty and EPDD on effort exertion and subjective flow experiences, we also collected data on flow-related brain activity. Building on previous studies (Lu et al., 2023; Van der Linden et al., 2021a; Linden et al., 2021b), we focused on the P300 component of EEG measures. The P300 is an event-related potential (ERP) elicited by task-relevant stimuli, typically peaking around 300 milliseconds after stimulus onset (Picton, 1992). Research has suggested that the P300 serves as a valuable physiological marker for assessing task engagement and flow-related experiences because of their shared relevance with the locus coeruleus-norepinephrine (LC-NE) system (Lu et al., 2023; Murphy et al., 2011; Van der Linden et al., 2021b). Notably, studies have shown that the amplitude of the stimulus-evoked P300 follows an inverted U-shaped pattern across varying levels of task difficulty, mirroring the pattern observed in subjective reports of flow experiences (Lu et al., 2023; Murphy et al., 2011). Further supporting this connection, studies on effort exertion have demonstrated that the P300 amplitude tends to be larger under conditions of high effort, suggesting a link between effort levels and P300 responses (Bowyer et al., 2021; Zhang & Zheng, 2022). These findings seem to indicate that the mixed effect of task difficulty and effort exertion may not only influence flow experience, but also modulate P300 responses. Based on this evidence, we hypothesize that the P300 will exhibit a trajectory similar to that of flow experiences, reflecting changes in task difficulty and effort exertion.

It is worth noting that both P300 and pupil diameter have been proposed as indicators of activity in the LC-NE system, which is implicated in flow states (Lu et al., 2023). However, the visual discrimination task employed in this study posed challenges for using pupil diameter as a reliable measure (Mathôt et al., 2018). Specifically, the task’s prolonged processing times interfered with the temporal dynamics of pupil dilation, making it difficult to isolate task-specific responses. Moreover, luminance changes and frequent saccades during the task introduced noise unrelated to flow or effort exertion, further complicating the interpretation of pupil data. In contrast, the P300 offered a more robust and task-specific measure for examining flow-related brain activity within this context.

In summary, we propose the following key hypotheses: First, we posit that the relationship between the flow state and task difficulty follows an inverted U-shaped curve, where optimal flow is experienced when task difficulty is well-matched to an individual’s skill level. Second, we hypothesize that a higher EPDD motivates individuals to exert greater effort. Third, we anticipate that this increased effort exertion will positively influence the experience of flow. Specifically, we suggest that when individuals engage in a challenging task, the heightened effort they invest is accompanied by an enhanced flow experience. This effect is expected to amplify the right tail of the inverted U-shaped relationship between task difficulty and flow. Finally, building on evidence that stimulus-evoked P300 serves as a potential physiological marker of the flow state, we predict that the P300 amplitude will vary systematically as a function of the interplay between task difficulty, effort exertion, and flow experience.

## Methods

### Participants

The study was conducted in accordance with the ethical guidelines of the MASKED University. Informed consent was obtained from all participants. Thirty-nine undergraduate students were recruited from the university. One participant was excluded due to the loss of EEG data. Another participant was excluded because their reported personal experience (Refer to flow experience measure procedure being described below) didn’t align with the characteristics of flow, indicating a misunderstanding of the concept. The final sample size was 37 (29 females, age = 19.87±1.78). All participants included in the final sample were well-rested and in good health, with normal or corrected-to normal vision.

### Experimental design

A 3 × 2 within-subject experimental design was employed to examine the effects of objective task difficulty (easy, intermediate, difficult) and EPDD (CE = control expectancy; HE = high expectancy) on subjective task difficulty, effort exertion, perceived flow, and relevant EEG indicators. Participants performed a visual discrimination task, in which they were asked to determine whether two simultaneously presented images were the same or different. Visual discrimination is a perceptual task that requires focused attention and involves actively scanning the visual environment for specific features. The images used in the experiment were composed of a grid of black and light gray squares. In each trial, the two images displayed either the same or have one black square shifted its position. The position of the differing square was randomly assigned across trials.

The difficulty level of the task was manipulated by varying the stimulus complexity, defined by the number of elements participants needed to scan within a fixed time frame. Specifically, the images were composed of a grid of black and gray squares: 2 × 2 squares for the easy condition, 8 × 8 squares for the intermediate condition, and 14 × 14 squares for the difficult condition.

The EPDD was manipulated by informing participants of the expected percentage of trials with different images in the upcoming block. In the CE conditions, participants were told that the images would differ in 50% of the trials, which reflected the actual ratio of same-to-different trials. In the HE conditions, participants were informed that the probability of the images being different was 80%, representing an exaggerated ratio. The specific percentages used for the EPDD manipulation were determined based on fine-tuning during pilot testing to ensure they effectively influenced participants’ expectations. The pilot testing focused on comparing a control condition (50% difference) with an increased difference condition to determine the optimal balance between eliciting meaningful changes in reaction time and ensuring participants remained unaware of the manipulation. The results guided the researchers to use the 80% EPDD as a high expectancy in the main study, as it achieved the desired effects without compromising the study’s validity.

### Procedure

**Fig. 1:**
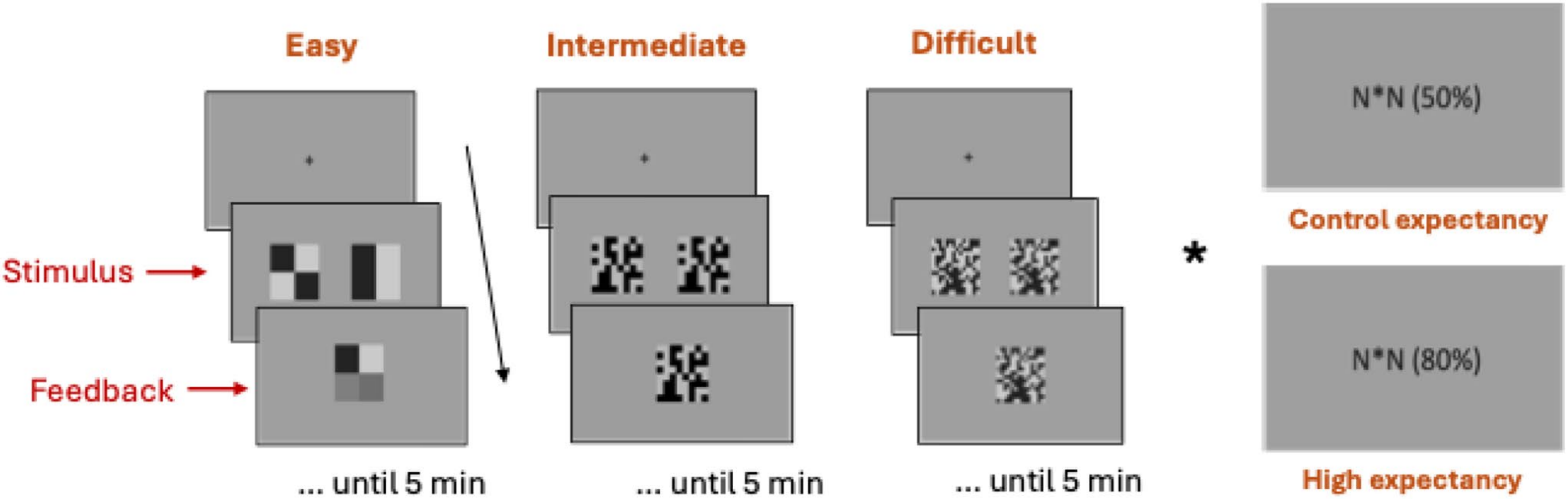
Illustration of the experimental design. N*N represents the number of squares of each image in the following block. 50% represent 50% difference probability; 80% represent 80% difference probability

After providing their informed consent, participants were given a short description of the flow experience – followed by a paper-based interview asking them to write down one of their past flow experiences. Then, participants performed the visual discrimination task on a computer with continuous EEG recorded during the task. The experimental task was programmed using the E-prime software and presented on a screen with resolution of 1920 × 1080 pixels and refresh rate of 120 Hz. The task consisted of six blocks (easy & CE, easy & HE, intermediate & CE, intermediate & HE, difficult & CE, difficult & HE; see Fig. 1). Participants completed the six task blocks in random order. Each block lasted for 5 min. Trials in each session started with a fixation cross lasting for 1s, followed by the stimuli. Participants were expected to make the decision of whether the two images were the same (press button ‘F’) or not (press button ‘J’) within 5s. After a response was given, a blank screen was displayed for 1.5 s, followed by an overlay image of the two previously shown images. The differences were highlighted in gray, and if the images were identical, no gray highlights were present. The overlay image was shown for 1s. After each block, participants reported their perceived flow experience and perceived challenge level.

With a 5-minute time limit for each block, the experiment resulted in varying numbers of trials across blocks. The average number of trials across the six conditions were as follows: 47.6 ± 6.33 for easy & CE condition, 47.8 ± 6.56 for easy & HE condition, 37.4 ± 5.65 for intermediate & CE condition, 36.9 ± 5.82 for intermediate & HE condition, 34.1 ± 6.30 for difficult & CE condition, and 33.7 ± 6.30 for difficult & CE condition.

### Measures

#### Flow experience

Measuring subjective flow poses a persistent challenge in flow studies, primarily due to its abstract and complex nature, making it difficult to capture it effectively using existing scales with multiple items (Biasutti et al., 2018; Lu et al., 2023). For instance, Lu et al.’s (2023) study reported that internal consistencies when utilizing a multiple-item scale is poor in terms of consistency. To address this issue comprehensively, the current study adopted a novel approach by combining interviews and questionnaires. This methodology aimed to inspect participants’ understanding of the flow concept before instructing them to report their flow levels during the experiment. The advantage of this mixed method has been reported in previous studies (Harris & Brown, 2019; Reams & Twale, 2008; Yafele, 2022).

In order to inspect participants’ understanding of the flow state, a paper-based semi-structured interview was conducted before the experiment. The instruction was as follows, ‘*In positive psychology*, *a flow state*, *also known colloquially as ‘being in the zone’*, *is the mental state in which a person performing some activity is fully immersed*, *has full involvement*, *and enjoyment in the process of the activity. In essence*, *flow is characterized by the complete absorption in what one does*, *and a resulting transformation in one’s sense of time. Have you ever experienced flow in your work/study/sports/leisure time? Please recall a scenario you have experienced that you think is most close to the flow state. (e.g. what kind of activity were you doing when you had that feeling? How did you feel? Did you try to get into that state again? Did you succeed? What kind of activities were more likely to trigger a flow state for you?)*. The background helped explore their personal interpretations and experiences related to flow. The interview served as groundwork to identify any misunderstandings participants may have had regarding the flow state. After screening the report content, one participant was excluded because their report was identified as non-flow experience by an expert (the original data could be found via https://surfdrive.surf.nl/files/index.php/s/5enlx1vuLQWKtDc). By incorporating the paper-based interview phase before administering the scale, we sought to establish a robust foundation for participants’ understanding of flow, ensuring that responses from all included participants reflected a more precise and coherent comprehension of the concept. This approach aimed to mitigate potential misinterpretations of the abstract items and improve the accuracy and validity of the flow measurements.

Based on the preceding methodology, during the task, we measured flow by asking ‘To what extent do you think you were in a flow state?’ using a 10-point scale (1 = not at all, 10 = very much; (Dawes, 2008). By utilizing this direct measurement, we aimed to efficiently gauge participants’ perceived level of flow without burdening them with an extensive scale, while still providing valuable insights into their flow experiences. The use of this single-item scale in conjunction with the interview phase ensures a more comprehensive understanding of flow, facilitating a more accurate and meaningful examination of the flow phenomenon across diverse contexts and domains.

#### Perceived task difficulty

*Perceived task difficulty* was measured after each block by asking ‘To what extent do you believe the task matches your personal skill?’. A 7-point scale was used (-3 = much lower than my skill, 0 = match, 3 = much higher than my skill).

#### Effort exertion

The amount of effort people put into a task is usually being encoded as the amount of cognitive resources and the amount of time they put in it (Hiemstra et al., 2019; Kellogg, 1987; Pollart et al., 2015). Accordingly, and similar to previous studies (Gheza et al., 2023), the amount of time spent on finding the difference in each trial and the correct rate in the current experimental setting were used as indicators of effort exertion. A longer time and/or a higher accuracy rate indicate a higher effort exertion.

#### Continuous EEG

*Continuous EEG* was recorded using a Biosemi ActiveTwo system. Sixty-four electrodes used in this study were mounted in an elastic cap using the international 10/20 system sites. A common mode sense (CMS) electrode is located near the PO1, and a driven right leg (DRL) electrode located near the site PO2. The horizontal electrooculogram (EOG) was recorded from electrodes placed lateral to the external canthi and was used to detect horizontal eye movements; vertical EOG recorded from two electrodes places above and below the left eye were used to detect eyeblinks and vertical eye movements. In line with BioSemi guidelines and practice from previous studies, DC offset < 20 mV were used as the standard to indicate good electrode contact (Lu et al., 2023; http://www.biosemi.com/publications/artikel3.htm). Online signals were recorded with a sample rate of 512 Hz and 24-bit A/D conversion.

### Data processing and statistical analysis

EEG signal processing was performed in Python using the MNE package. Bad channels were identified via visual inspection and interpolated using the spherical spline method. Raw data was filtered with a 0.1–30 Hz bandpass and referenced to the computed average. We then compute independent components using FastICA (Hyvarinen, 1999) on a duplicated continuous data high pass filtered by 1 Hz and applied the result to the original 0.1 Hz filtered data. Sixty-three components were extracted and being inspected manually in a conservative strategy (less than 4 out of 63 components were removed, mainly eye components and clear muscle components). Epochs were then segmented beginning 200ms prior to the onset of the stimulus and continuing for 1000ms post stimulus, baseline correction was performed using the 200ms prior to stimulus onset. Epochs with abnormal values exceed ±150 µV or flat over 5s were excluded from further analysis. Stimulus-evoked P300 was calculated as the mean value between 300ms and 400ms on the Pz electrode across all valid trials in each condition. Both correct and incorrect trials were included in the calculation of P300. The number of trials included in the final analysis for all conditions are different due to the self-paced nature of the task and the data quality variations (easy & CE = 45.24±2.17, easy & HE = 45.22±2.62, intermediate & CE = 34.97±3.55, intermediate & HE = 34.59±2.98, difficult & CE = 31.84±2.97, difficult & HE = 31.86±2.57).

Preprocessed data were further analyzed using R 4.1.2 and visualized using R and python. Given the exploratory nature of several operations and measures in this study, we conducted a series of manipulation checks to ensure the effectiveness of our experimental manipulations.

To examine the effects of our manipulations on perceived task difficulty, we employed mixed-effects modeling. Our goal was to create two effort levels across three difficulty conditions (easy, intermediate, and difficult) without altering perceived task difficulty. As such, we expected a significant main effect of the task difficulty manipulation but no significant main effect of the EPDD manipulation on perceived task difficulty. To further validate that the task conditions captured a sufficient range of perceived difficulties—ranging from underload to challenge-skill match, to overload— we conducted one-sample t-tests on participants’ reported challenge levels across the six conditions: easy & CE, easy & HE, intermediate & CE, intermediate & HE, difficult & CE, and difficult & HE. These tests used the “match” anchor (0 on a -3 to 3 scale) as the baseline. Reported challenge levels significantly lower than the match anchor indicated that participants perceived the task as easier than their ability, while levels significantly higher than the match anchor indicated the task was perceived as more difficult than their ability. Levels not differing from the match anchor reflected intermediate difficulty that matched participants’ abilities.

Effort exertion was evaluated using mixed-effects modeling, with reaction time (RT) and accuracy rate serving as key indicators. Trials with RTs shorter than 100 ms were excluded as outliers, while all other trials—both correct and incorrect—were included in the analysis. Due to a 5-second response limit, any responses exceeding this time were considered incorrect. We hypothesized that both task difficulty and EPDD manipulation would significantly influence effort exertion, predicting main effects of both factors on RT and accuracy. These analyses confirmed that our experimental manipulations effectively elicited the intended variations in effort, validating the design of the study.

Following the manipulation checks, we tested the effects of task difficulty and EPDD manipulation on flow and flow-related EEG indicators (e.g., P300 amplitude). We first evaluated whether flow and P300 amplitude exhibited a classic inverted U-shaped pattern across task difficulty. Mixed-effects modeling was conducted to test the quadratic trend for each EPDD condition, providing insights into how flow changes with task difficulty under varying EPDD conditions. To further explore the interplay between task difficulty and EPDD manipulation, we used mixed-effects modeling to assess the main effects and interaction effects of these factors on flow. These analyses allowed us to understand the finer dynamics of how task difficulty and EPDD manipulation jointly influence flow.

All mixed-effects models included subject ID as a random intercept to account for individual differences. The models were implemented using the lme4 package (Bates et al., 2015). Post-hoc tests were conducted using the emmeans package (https://CRAN.R-project.org/package=emmeans), with p-values adjusted for multiple comparisons using the Tukey method. Degrees of freedom for simple effects tests were estimated using the Kenward-Roger method (Chawla et al., 2014).

To ensure robust and interpretable results, we reported effect sizes for all analyses. For t-tests, we used Cohen’s d, which provides a standardized measure of the difference between means. In the current study, the absolute values of observed Cohen’s d ranged from 0.01 (very small effect) to 2.3 (very large effect), corresponding to power estimates ranging from low (0.05) to very high (> 0.99). For mixed-effects models testing main effects and interactions, we reported partial eta squared (*ηp²*) to quantify the proportion of variance explained by each predictor. The reported *ηp²* values ranged from < 0.001 (very small effect) to 0.64 (very large effect), yielding power estimates ranging from low (0.06) to very high (> 0.99). For models examining the inverted U-pattern, we reported conditional R² (variance explained by both fixed and random effects) and marginal R² (variance explained by fixed effects only). In this study, conditional R² ranged from 0.214 to 0.879, while marginal R² ranged from 0 to 0.028. The moderate to high conditional R² values indicate that the inclusion of random effects substantially improves the model’s explanatory power, whereas the low marginal R² values suggest that fixed effects alone explain very little variance in the outcome. These measures were chosen based on established practices in the literature (e.g., Lakens, 2013; Nakagawa & Schielzeth, 2013). Cohen’s d was selected for t-tests due to its interpretability as a standardized measure of effect size, while partial eta squared (*ηp²*) was used for ANOVA to quantify the proportion of variance explained by each predictor. Conditional and marginal R² were reported for mixed-effects models to distinguish the contributions of fixed and random effects, consistent with recommendations for complex models.

## Results

### Perceived task difficulty

**Fig. 2:**
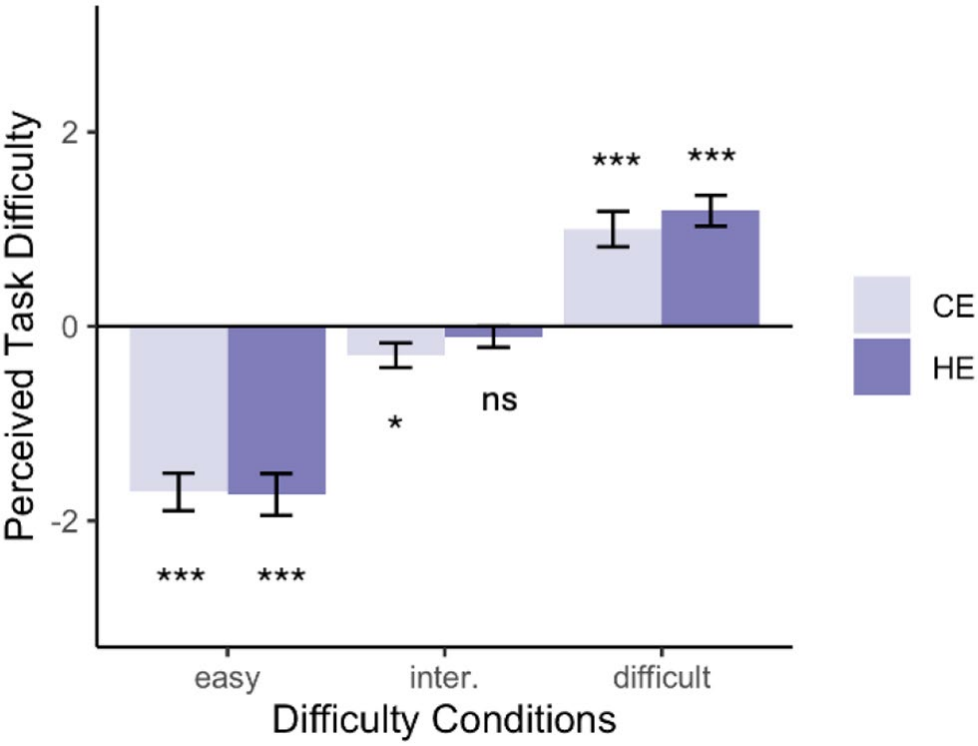
Self-reported challenge level across three pre-defined difficulty conditions and two difference probability expectancy conditions (CE = control expectancy, HE = high expectancy)

Results from the mixed-effects modeling on the conditional level revealed a significant main effect of the task difficulty manipulation on perceived task difficulty (Fig. 2, F(180, 2) = 162.80, *p* < 0.001, *η*_*p*_^*2*^ *=* 0.64), indicating that the manipulation was successful. In contrast, there was no significant main effect of EPDD on perceived task difficulty (F(180, 1) = 0.85, *p* = 0.36, *η*_*p*_^*2*^ < 0.001), confirming that EPDD did not independently affect participants’ difficulty perceptions. Furthermore, the interaction between task difficulty manipulation and EPDD was not significant (F(180, 2) = 0.32, *p* = 0.73, *η*_*p*_^*2*^ = 0.004), showing that the effect of task difficulty manipulation did not depend on participants’ EPDD. Post hoc pairwise comparisons revealed significant differences in perceived task difficulty between all levels of difficulty conditions (all *ps* < 0.001). Specifically, participants reported significant lower perceived task difficulty in easy condition than intermediate condition (*t*(180) = 9.71, *p* < 0.001, Cohen’s *d* = 1.24) and difficult condition (*t*(180) = 18.03, *p* < 0.001, Cohen’s *d* = 2.30). Participants also had significantly low perceived task difficulty in intermediate condition compared to difficult condition (*t*(180) = 8.32, *p* < 0.001, Cohen’s *d* = 1.07).

One sample t-tests were conducted to compare participants’ reported challenge levels to a ‘match’ anchor (0 on a -3 to 3 scale) across the six conditions. Results revealed significant differences in most conditions. In the easy conditions, participants reported significantly lower challenge levels (CE: M = -1.70, *t*(36) = 8.81, *p* < 0.001, Cohen’s *d* = 1.45; HE: M = 1.73, *t*(36) = 8.06, *p* < 0.001, Cohen’s *d* = 1.32), indicating that they perceived the task as much easier than their ability. In the intermediate & CE condition, participants reported challenge levels that were slightly lower than the baseline (CE: M = -0.30, *t*(36) = 2.33, *p* = 0.026, Cohen’s *d* = 0.38). However, as expected, in the intermediate & HE condition, participants reported challenge levels that did not differ significantly from the baseline (HE: M = -0.11, *t*(36) = 1.00, *p* = 0.324, Cohen’s *d* = 0.16), suggesting that the task was perceived as neither easier nor more challenging than the anchor. In the difficult conditions, participants reported significantly higher challenge levels than their ability (CE: M = 1.00, *t*(36) = 5.50, *p* < 0.001, Cohen’s *d* = 0.90; HE: M = 1.19, *t*(36) = 7.48, *p* < 0.001, Cohen’s *d* = 1.23). These results suggest that the experiment captures individuals’ perceived task difficulty from easy to difficult levels and captures a level of which difficulty matched with individuals’ ability.

### Indicators of effort exertion

**Fig. 3:**
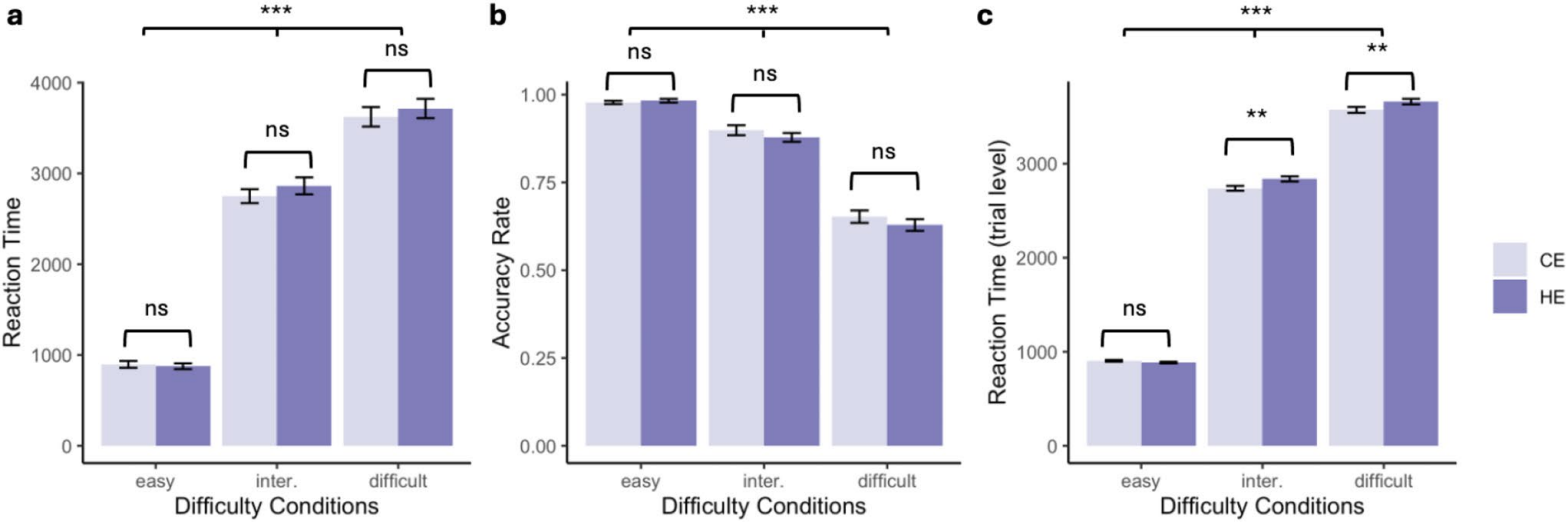
**a**) Reaction time and **b**) accuracy rate in three pre-defined difficulty conditions and two difference probability expectancy conditions (CE = control expectancy, HE = high expectancy). Comparisons are on individual level. **c**) Trial level reaction time comparisons in difficulty and expectancy conditions

Reaction time and accuracy rate were used as indicators of effort exertion. Results from the mixed-effects model on the conditional level revealed a significant main effect of task difficulty on reaction time (F(180,2) = 1202.45, *p* < 0.001, *η*_*p*_^*2*^ = 0.93). However, there was neither a main effect of EPDD (F(180,1) = 1.69, *p* = 0.195, *η*_*p*_^*2*^ < 0.01) nor an interaction effect between task difficulty and EPDD (F(180,2) = 0.76, *p* = 0.468, *η*_*p*_^*2*^ < 0.01). Post hoc pairwise comparisons confirmed that reaction time increased significantly with increasing task difficulty, with significant differences between all task difficulty conditions (all *ps* < 0.001; see Fig. 3a).

For accuracy rate, results from the mixed-effects model revealed a significant main effect of task difficulty (F(180, 2) = 533.53, *p* < 0.001, *η*_*p*_^*2*^ = 0.86). However, again, there was no significant main effect of EPDD (F(180, 1) = 2.23, *p* = 0.137, *η*_*p*_^*2*^ = 0.01), and no significant task difficulty × EPDD interaction effect (F(180, 2) = 1.05, *p* = 0.351, *η*_*p*_^*2*^ = 0.01). Post hoc pairwise comparisons confirmed that accuracy decreased significantly between all ascending task difficulty conditions (all *ps* < 0.001; see Fig. 3b).

To further investigate the effects of EPDD on effort exertion, we conducted additional exploratory analyses at the trial level, which provided greater sensitivity to detect subtle effects due to the larger number of observations (*N* = 8785). Trial-level analysis captures real-time adjustments in effort and decision-making as participants respond to varying task demands and expectations. In contrast, individual-level analyses aggregate data across trials, potentially masking these nuanced variations. Since difference location on images varied randomly across trials in each condition, participants exerted more effort in trials with greater uncertainty. The trial-level analyses were conducted on reaction time. Accuracy rate cannot be calculated on a trial-by-trial basis, because it is inherently a cumulative measure derived from multiple trials. Results are visualized in Fig. 3c. The trial-level analyses revealed a significant main effect of task difficulty (F(2, 8743.3) = 10549.40, *p* < 0.001, *η*_*p*_^*2*^ = 0.71) and EPDD (F(1, 8743.1) = 11.93, *p* < 0.001, *η*_*p*_^*2*^ < 0.01) on reaction time. Additionally, there was a significant interaction effect between task difficulty and EPDD (F(2, 8743.1) = 6.10, *p* = 0.002, *η*_*p*_^*2*^ < 0.01).

Post hoc pairwise comparisons demonstrated that all three difficulty conditions were significantly different from each other in both EPDD conditions (all *ps* < 0.001). Furthermore, comparisons between EPDD conditions within each difficulty level revealed some significant differences. Specifically, for the easy task, there was no difference in reaction time between the control and high expectancy conditions (*t*(8743) = 0.78, *p* = 0.436, Cohen’s *d* = 0.01). In contrast, for the intermediate and difficult tasks, the high expectancy condition led to significantly longer reaction times compared to the control expectancy condition (intermediate task: *t*(8743) = 3.50, *p* = 0.005, Cohen’s *d* = 0.05; difficult task: *t*(8743) = 2.90, *p* = 0.003, Cohen’s *d* = 0.04). These findings suggest that participants exerted greater effort on trials when they had a higher expectancy of difference probability, particularly in more difficult tasks. Notably, this effect was observed at the trial level but not at the individual level, indicating a small but meaningful effect that required the increased sensitivity of trial-level analysis to detect. Given the post hoc nature of these trial-level effects, these analyses should be interpreted with caution because they are exploratory.

### Perceived flow

Overall, the visual discrimination task induced moderate to high flow experience (M = 6.14 on a ten-point scale, SD = 1.977). We tested the inverted U-pattern of perceived flow across Task Difficulty in both expectancy conditions by fitting quadratic models. A marginal significant quadratic pattern was observed for the control expectancy condition (*b*_*(difficulty*_^*2*^_*)*_ = -0.66, *p* = 0.053, B = -0.35, Conditional R^2^ = 0.214, Marginal R^2^ = 0.028). No quadratic pattern was observed for the high expectancy condition (*b*_*(difficulty*_^*2*^_*)*_ = -0.35, *p* = 0.292, B = 0.16, Conditional R^2^ = 0.388, Marginal R^2^ = 0.009). This provides preliminary evidence for a changed trend in flow experience in the high (vs. control) expectancy condition.

**Fig. 4:**
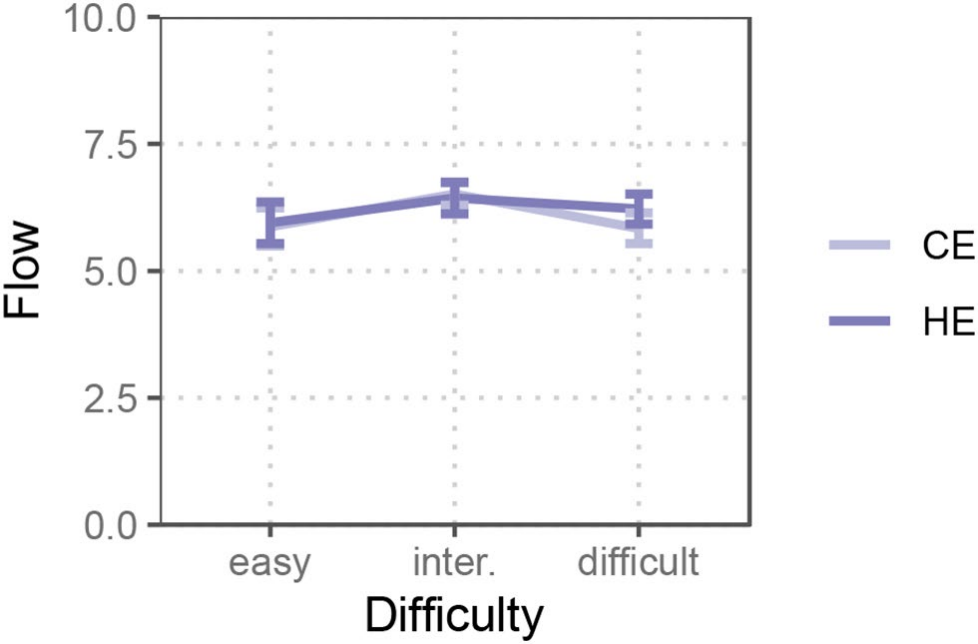
Self-reported flow experience in three pre-defined difficulties and two target expectancy conditions (CE = control expectancy, HE = high expectancy)

Further results from the mixed effect modeling revealed a weak interplay between task difficulty and EPDD on flow. Specifically, we found that the main effect of task difficulty approached statistical significance (F(2, 180) = 2.63, *p* = 0.074, *η*_*p*_^*2*^ *= 0.03*). There was neither a main effect of EPDD (F(1, 180) = 0.35, *p* = 0.554, *η*_*p*_^*2*^ *< 0.01*), nor a task difficulty × EPDD interaction effect (F(2, 180) = 0.40, *p* = 0.670, *η*_*p*_^*2*^ *< 0.01*). Further simple effect tests revealed no differences between conditions. Even though Fig. 4 shows a slightly leveraged right tail of the trend in the high expectancy condition, the increase in flow experience failed to reach significance (*t(36)* = 1.69, *p* = 0.099, Cohen’s *d* = 0.28). Means and standard errors of the flow experience in each condition are visualized in Fig. 4.

### Stimulus-evoked P300 amplitude

**Fig. 5:**
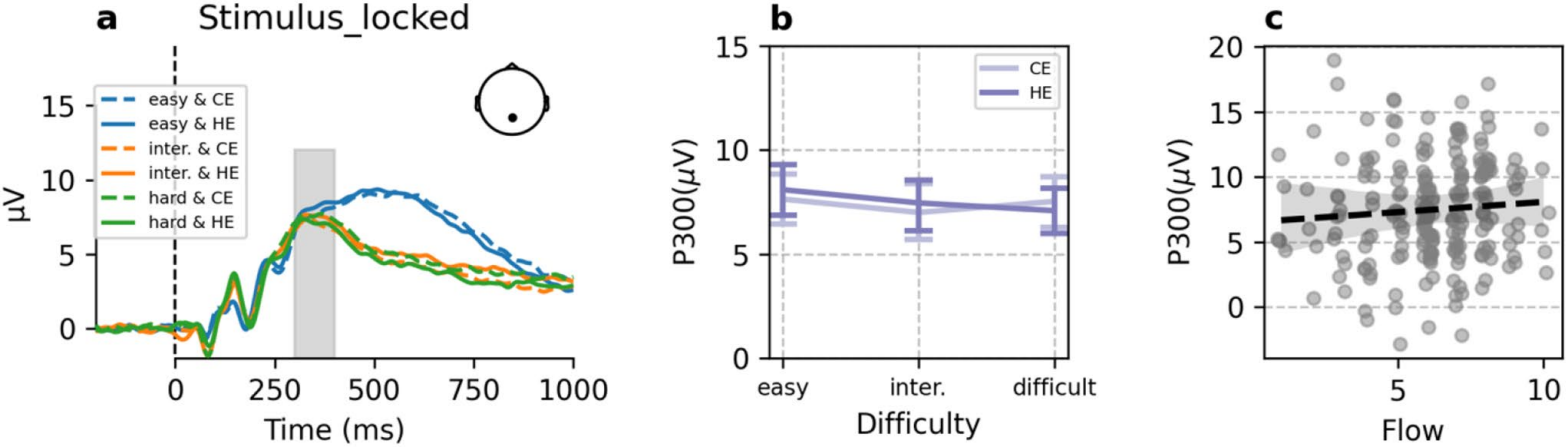
**a**) Grand averaged ERP waveform (stimuli-locked) on the Pz electrode in six experimental conditions. P300 were extracted from the time window between 300 and 400ms. **b**) stimulus-evoked P3 amplitude in different difficulty and EPDD conditions. **c**) The relation between flow and P300 amplitude

Grand averaged continuous EEG waveform locked with stimulus in Pz electrode is shown in Fig. 5a. We hypothesized that stimulus-evoked P300 amplitude would follow the same trajectory as the flow experience in different experimental conditions. We fitted the P300 data in both target expectancy groups with a quadratic model. The results show that both groups failed to fit the quadratic trend across increasing task difficulty (CE: *b*_*(difficulty*_^*2*^_*)*_ = 0.13, *p* = 0.706, B = 0.15, Conditional R^2^ = 0.800, Marginal R^2^ = 0.012; HE: *b*_*(difficulty*_^*2*^_*)*_ = 0.58, *p* = 0.140, B = 0.03, Conditional R^2^ = 0.763, Marginal R^2^ = 0.005, see Fig. 5b).

Subsequently, we tested the relationship between the flow scores and the P300 amplitude using mixed effect model with subjective number entered as random intercept, task difficulty and EPDD entered as fixed slope. The results suggested that there is no direct relationship between flow and the stimulus-evoked P300 amplitude (*b*_*(flow)*_ = -0.003, *p* = 0.97, B < 0.001, Conditional R^2^ = 0.879, Marginal R^2^ = 0.000, see Fig. 5c).

## Discussion

Investigating the interplay between task difficulty and effort poses a significant challenge due to the inherent difficulty of disentangling the two constructs. This challenge arise because, as per energy conservation theory, alterations in effort exertion tend to closely correspond with task difficulty or demand (Hobfoll, 1989). Based on the expected value of control theory (Shenhav et al., 2013), we introduced the EPDD manipulation aimed to induce variations in effort without changing the actual task difficulty. In this study, we hypothesized that flow-like feelings across varying levels of task difficulty might be associated with changes in effort exertion. Several hypotheses were tested to explore the intricate relationship between task difficulty, effort exertion, and the experience of flow. Our findings provide partial support for these hypotheses and offer new insights into the mechanisms underlying flow.

First, we proposed and confirmed that our task difficulty manipulation successfully captured the skill-challenge match condition, as well as the underload and overload conditions. This validation was critical for ensuring that our experimental design effectively represented the continuum of task difficulty as theorized in flow literature (Csikszentmihalyi, 1997). Specifically, this approach allowed us to operationalize key theoretical constructs of flow theory, where optimal experiences are theorized to particularly emerge when skills and challenges are in balance, and to contrast them with underload and overload conditions, respectively.

Second, we found that the high expectancy condition resulted in longer response times, particularly during intermediate and difficult tasks. This increase in reaction time was interpreted as reflecting greater effort exertion in these tasks, suggesting that EPDD may modulate effort allocation. Specifically, participants appeared to exert more effort when they anticipated a higher likelihood of detecting differences between images. This finding aligns with the notion that individuals adjust their effort based on their expectations of task outcomes (Frömer et al., 2021; Shenhav et al., 2013). However, it is relevant to note that this effect was observed only at the trial level, with no significant effect detected at the individual level. This suggests that the effect size may be relatively small, and the results should be interpreted with caution. Alternatively, it may indicate that the phenomenon primarily manifests at the trial level. Given that the different squares in the task images were randomly assigned, some trials may have required less scrutiny to detect differences. For instance, effort exertion may be more strongly influenced by expectation in trials with greater uncertainty, where participants are required to engage more cognitive resources to resolve ambiguity. Additionally, the absence of a manipulation effect in the easy task are in line with the notion that in these tasks, detecting differences is directly obvious, and exerting additional effort would not be useful (Gibson, 1900; Silvestrini et al., 2023).

Third, for difficult tasks, we identified a trend suggesting slight increase in flow in the high expectancy condition compared to the control condition. Further analyses, however, did not reveal a significant interaction between task difficulty and EPDD. Specifically, in the control condition, we indeed found an inverted U pattern of flow feelings corresponding to increasing task difficulty, consistent with flow theory and the optimal challenge assumption (Csikszentmihalyi & Lefevre, 1989). This pattern indicates that flow is most likely to occur when task difficulty aligns with an individual’s skill level. Interestingly, in the high expectancy condition, we failed to detect such an inverted U-pattern of flow. It seems that the slightly higher flow, evoked by difficult tasks may have prevented us from identifying an inverted U-pattern. Overall, the pattern of findings, including the tendency towards more flow in the high expectancy condition, indirectly supports our idea that flow may not be determined solely by task difficulty, but also by the amount of effort an individual is willing to invest in a task. This suggests that effort investment, potentially driven by expectancy or motivation, could play a role in shaping flow experiences. For example, recent work demonstrated that the process that learning progress guiding engagement and cognitive effort (e.g., proactive preparation, feedback processing) may serve as important basis for flow (Lu et al., 2025).

While we observed an inverted U-shaped pattern between task difficulty and flow, we did not find a similar pattern between task difficulty and reaction time indexed effort. Instead, reaction times increased consistently as tasks became more difficult. This outcome is expected, as participants naturally required more time to respond as task complexity grew. Thus, while the subtle differences in reaction time between the high expectancy condition and the control condition likely reflected additional effort (e.g., prolonged search even when difficulty remained constant), across the three levels of task difficulty, reaction times primarily mirrored the overall effort (search time) driven by increasing task complexity. Alternatively, this result may stem from the complex dynamics of effort regulation, where task difficulty dominates when tasks are perceived as achievable (Hopstaken et al., 2015; Richter et al., 2016). By introducing a EPDD manipulation, we demonstrated that effort exertion increases in response to difficult tasks under high expectancy conditions, aligning with corresponding changes in flow. This indirect evidence suggests that effort may play a role in facilitating flow under certain motivational and cognitive conditions, even if its relationship with task difficulty does not follow a straightforward inverted U-shaped pattern. While these results provide intriguing insights, further research is needed to investigate the nuanced interplay between effort, task difficulty, and flow using direct and systematic approaches.

Finally, we found that P300 amplitude was independent of flow feelings, challenging assumptions about the neural correlates of flow. The divergence between our anticipated pattern and the observed findings in P300 introduces intriguing complexities in understanding the relationship between effort, task difficulty, and the neural underpinnings of flow. It is relevant to note that this is not the only study reporting a null association between flow and P300. A similar null effect was also reported in previous research (LoTemplio et al., 2021; Lu et al., 2023). This discrepancy prompts us to reconsider existing models that assume a straightforward alignment between the P300 indexed phasic LC-NE activity and flow dynamics (Van der Linden et al., 2021b). This opens avenues for more refined investigations into the neural underpinnings of flow, acknowledging the intricate and context-dependent nature of these relationships.

The present study represents an exploratory effort to uncover the nuanced relationship between effort exertion and subjective flow, offering initial but innovative insights into how these factors might interact. While the findings provide a foundation for understanding the role of effort in fostering flow, the small effect sizes and limitations in the manipulation design underscore the need for further investigation. The present study highlights the complexity of these interactions and emphasizes the importance of refining experimental approaches to better capture the underlying mechanisms.

One potential explanation that the findings on reaction time indexed effort and flow are subtle in this study is the relatively weak effort manipulation as indicated by relative low effect sizes. Although the effect of EPDD on effort exertion was evident at the trial level, it was not significant at the individual level, indicating a limited influence of the EPDD. To achieve stronger effects in future research, several aspects of the experimental design could be refined. For instance, the expectancy manipulation in this study was implemented at the block level, which may have diluted its impact on trial-by-trial responses. Possibly, a trial-level manipulation of expectancy could maximize its influence and yield more pronounced results. Additionally, the EPDD conditions in this study were both relatively high (CE = 50% and HE = 80%), lacking a lower expectancy condition. Given that participants in experimental settings often exhibit a natural tendency to perform well, even in control conditions, this may have created a ceiling effect on effort exertion, obscuring potential differences. Introducing a low expectancy condition in future studies could help address this limitation and provide a more nuanced understanding of the relationship between expectancy and effort.

The modest sample size in this study is a limitation to take into account when interpreting the findings. The sample size was determined primarily based on practical considerations (Simmons et al., 2011), and while it allowed us to explore the effects of task difficulty and effort exertion on flow, it is associated with a lower power to detect effects. Future research would benefit from conducting a priori power calculations to ensure adequate sample sizes, thereby enhancing the robustness and generalizability of the findings (Cohen, 1992).

Moreover, while reaction time has been widely used as a proxy for effort allocation in prior research (e.g., Hiemstra et al., 2019; Kellogg, 1987), relying solely on this measure may be suboptimal. Reaction time is susceptible to a range of factors, including individual differences in cognitive processing speed, levels of motivation, and states of fatigue (Cohen, 2014; Prabu Kumar et al., 2020), suggesting that a multifaceted approach to gauging effort is more appropriate. Future studies should consider integrating multiple indicators such as physiological measures (e.g., heart rate variability), subjective self-reports, and performance metrics to achieve a more comprehensive and accurate assessment of effort (Bahameish & Stockman, 2024; Thayer et al., 2009).

Another important finding that warrants further exploration is the dissociation between reaction time and accuracy. While participants in the high-expectancy (HE) condition exhibited longer reaction times—particularly in intermediate and difficult tasks—this increased processing duration did not translate into improved accuracy. This pattern suggests that both expectancy groups were equally capable of detecting differences and similarities in the matrices, as reflected in their comparable accuracy rates. However, under HE, participants engaged in prolonged verification processes before confirming their judgments. Additional analysis (presented in Supplementary Materials) revealed that HE selectively extended reaction times in two key scenarios: (1) during “no-difference” judgments (same-image trials) in difficult tasks, and (2) during “difference” judgments (different-image trials) in intermediate tasks. This suggests that heightened expectancy may differentially influence verification processes depending on task difficulty and judgment type. A similar dissociation between prolonged reaction times and unchanged accuracy has been observed in visual discrimination tasks, where extended processing reduced perceptual bias without improving accuracy (Dekel & Sagi, 2020), consistent with an evidence accumulation framework. However, these effects were modest and not uniformly observed across all conditions, indicating that the underlying mechanisms remain unclear. Future studies could employ process-tracing methods—such as eye-tracking or confidence ratings (Brunyé & Gardony, 2017)—to clarify whether HE prompts more thorough comparisons or heightened decision uncertainty.

The measurement of flow presents enduring challenges, as widely acknowledged in the literature (Cheron, 2016; Norsworthy et al., 2021). The abstract nature of flow makes it difficult to operationalize the construct. Traditional scales often assess multiple dimensions of flow, yet not all dimensions equally contribute to the flow experience. In our study, we introduced a novel approach by combining an interview with a questionnaire to facilitate a more direct and accurate assessment. The paper-based semi-structed interview served as a verification step, ensuring that participants had a clear understanding of the flow concept before completing the questionnaire. This approach helped improve the validity of self-reported flow measures by reducing potential misinterpretations. By refining the measurement process, we aimed to enhance the reliability of the flow assessment and contribute to more precise investigations of flow across various contexts.

While our innovative approach to measuring flow—combining interviews with questionnaires—represents a promising step forward in capturing subjective flow experiences, there are still areas for refinement. For example, the use of a single-item flow probing question on a 7-point Likert scale may not fully capture the nuanced variability inherent in the flow experience (Castro et al., 2023; Diamantopoulos et al., 2012). To improve the precision and sensitivity of flow measurement, future studies could adopt multi-item scales or expand the Likert scale to include more response options. Such enhancements would allow for a more detailed and accurate assessment of flow, better reflecting its complexity and variability across individuals and contexts.

Our finding that P300 amplitude was independent of flow feelings highlights the need to explore additional neural features in future studies to better understand the complexity of flow states. For instance, investigating frontal-midline theta oscillations could provide insights into cognitive control and task engagement, while alpha suppression in sensory regions might reveal mechanisms underlying attentional focus during flow (Biasutti et al., 2018; Lu et al., 2025; Sauseng et al., 2005). Similarly, examining dopaminergic activity in the striatum could shed light on the reward dynamics of flow, and reduced default mode network (DMN) activity may help clarify the neural basis of task immersion (Sayalı et al., 2023; Van der Linden et al., 2021a). Including these features in future research could provide a more comprehensive understanding of the multifaceted neural mechanisms driving flow experiences.

In summary, this study used a visual discrimination task to disentangle the effects of task difficulty and effort exertion on perceived flow. Our findings provide preliminary evidence for the intricate relationship between task difficulty, effort exertion, flow, and P300 amplitude. However, several aspects of the study design limit the strength of the conclusions that can be drawn. This underscores the need for further research to explore the complex interplay between task difficulty, effort exertion, flow, and associated neural mechanisms.

## Electronic supplementary material

Below is the link to the electronic supplementary material.

Supplementary Material 1

## Data Availability

Data that support the findings of this study can be found via https://surfdrive.surf.nl/files/index.php/s/5enlx1vuLQWKtDc.
