# Supplementary material for: Disentangling the effects of task difficulty and effort on flow experience
Source: Psychol Res. 2025 Jun 26;89(4):113. doi: 10.1007/s00426-025-02128-x (PMC12202627; doi:10.1007/s00426-025-02128-x)
Supplement: Supplementary file 1 — Supplementary Material 1 [file 426_2025_2128_MOESM1_ESM.docx]

**Disentangling the Effects of Task Difficulty and Effort on Flow Experience**

1. **Perceived task difficulty**

The result of manipulation effect of task difficulty and DP expectancy was further being confirmed by the results from the mixed effect modeling. We found that there was main effect of the objective task difficulty on perceived task difficulty (*F_(180,2)_* = 162.79, *p* < 0.001, *η_p_^2^* = 0.64) while no main effect of target expectancy (*F_(180,1)_* = 0.85, *p* = 0.359, *η_p_^2^* < 0.01) nor an interaction effect of task difficulty (*F_(180,2)_* = 0.32, *p* = 0.726, *η_p_^2^* <0.01). This indicates that the image complexity manipulation induced significant difference in perceived task difficulties, while the expectancy manipulation did not change the initial task difficulty.

1. **Effort exertion**

Results from the mixed effect model on the individual level revealed that, regarding reaction time and accuracy rate, there was significant main effects of task difficulty (RT: *F_(180,2)_* = 1202.45, *p* < 0.001, *η_p_^2^* = 0.93; ACC: *F_(180, 2)_* = 533.53, *p* < 0.001, *η_p_^2^ = 0.86*), but no main effects of the target expectancy (RT: *F_(180,1)_* = 1.69, *p* = 0.195, *η_p_^2^* < 0.01; ACC: *_F(180, 1)_* = 2.23, *p* = 0.137, *η_p_^2^ = 0.01*), nor an interaction effect between difficulty and target expectancy (RT: *F_(180,2)_* = 0.76, *p* = 0.468, *η_p_^2^ < 0.01*; ACC: *F_(180, 2)_* = 1.05, *p* = 0.351, *η_p_^2^ = 0.01*). Simple effect tests showed that the reaction time increased and accuracy decreased with increasing task difficulty (P<0.001 between all task difficulty conditions) see Figure S1a and S1b.

**Figure S1.** Reaction time (a) and accuracy rate (b) in three pre-defined difficulty conditions and two expectancy conditions (CE = control expectancy, HE = high expectancy).
